# COVID‐19 pandemic, vaccine nationalism and counterfeit products: Discourse and emerging research themes

**DOI:** 10.1002/tie.22302

**Published:** 2022-07-07

**Authors:** Joseph Amankwah‐Amoah, Robert E. Hinson

**Affiliations:** ^1^ Kent Business School University of Kent Kent UK; ^2^ Ghana Communication Technology University Accra Ghana

**Keywords:** business development, Covid‐19, COVID‐19 pandemic, vaccine diplomacy, vaccine nationalism

## Abstract

Although “vaccine nationalism” and vaccine diplomacy have thus far typified the COVID‐19 vaccine rollouts around the globe, there remain limited scholarly insights on global vaccine distribution strategies. This research note (RN) examines the global vaccine distribution strategies and implications for public policy and governments. In conceptualizing the global vaccine distribution strategies into three competing perspectives (i.e., “vaccine nationalism,” vaccine diplomacy, and global initiative), this article highlights the divergent effects of different approaches in terms of ushering elements of nationalism and ethnocentrism. By contextualizing the discourse on the COVID‐19 pandemic into the three competing perspectives and highlighting the role of pharmaceutical companies and COVID‐19 vaccine passport, the study also offers pathways for further examination of the subject incorporating the contextual conditions.

## 1 INTRODUCTION

Accompanying the COVID‐19 pandemic and governments' responses have been accelerating global inequality and “vaccine nationalism,” where the majority of early batches of vaccines have been largely assigned to developed and high‐income countries that often have much better healthcare and education systems compared with developing nations (Bollyky & Bown, 2020; Hassoun, 2021; Myre, 2020; Evenett, Hoekman, Rocha, & Ruta, 2021). Besides this, the pandemic and accompanying lockdown restrictions and border closures have culminated in “crisis for democracy around the world” (Repucci & Slipowitz, 2020, p. 1). According to Freedom House, some governments have capitalized on the pandemic as a pretext to suppress opposition, destabilize systems of accountability, control access to information, and marginalize minority groups (Repucci & Slipowitz, 2020). In just over a year, the COVID‐19 pandemic has ushered in unparalleled changes undercutting national economies and businesses (see Callaghan et al., 2021). Essentially, the pandemic has been typified by more than 501,234,520 coronavirus cases and 6,210,392 fatalities around the globe including more than 1,013,044 deaths in the United States alone and over 661,552 in Brazil (Worldometers, 2022). Although more than 451,450,136 individuals have contracted the virus and then recovered (Worldometers, 2022) and the pandemic has also inspired and accelerated technologies for remote working and touchless technologies at airports (Pande, 2020), the damage to national economies, political systems of government and businesses are apparent. For businesses, many organizational structures, business models, and processes have been rendered obsolete and many small and medium‐sized enterprises (SMEs) have closed permanently (Amankwah‐Amoah et al., 2021). The instances of “vaccine nationalism” have emerged as a counterforce to decades of pressures of globalization, where nations sought to ease restrictions to trade and investments. Indeed, some commentators have suggested that the nationalism force could erode much of the progress made in ushering in deregulation and liberalized markets (The Economist, 2020). As suggested by *The Economist* (2020, p. 7), “even before the pandemic, globalization was in trouble” with America's tariff rate on imports reverting to 1993 levels.

Although advancements in global deregulation and trade liberalization efforts have been crucial in ushering in 20th century globalization (Doganis, 2005; Ocampo & Taylor, 1998), these forces have come under sustained pressure in the 21st century not only from the pandemic but also from some global leaders who championed the notion of “nationalism” in their responses to it. According to the International Chamber of Commerce (2021), the global economy could lose up to US$9.2 trillion due to “vaccine nationalism” as governments have failed to ensure that developing economies gain equitable and timely access to COVID‐19 vaccines. Indeed, by late February 2021, the World Health Organization (WHO) observed that around 75% of the available vaccine doses were dispensed in around 10 wealthy nations (Blanchfield, 2021). Thus, for effective global economic recovery to occur, reliance on vaccines to mainly advanced economies would not yield robust economic growth until citizens of developing economies are also vaccinated. Despite new lines of research and discourse on the COVID‐19 pandemic, there is limited insight exploring global vaccine distribution strategies.

Against this backdrop, this research note (RN) examines the global vaccine distribution strategies and implications for public policy and governments. In view of the foregoing deficiencies in the current literature, the study makes several contributions to the current literature. First, in contrast with much of prior scholarly works on the COVID‐19 pandemic (e.g., Fernandes, Veiga, Lobo & Raposo, 2022; Santos, Oliveira, Ratten, Tavares & Tavares, 2021), this study conceptualizes global vaccine distribution strategies into three competing perspectives (i.e., “vaccine nationalism,” vaccine diplomacy, and global initiative), which provide not only deeper understanding of the current approaches but offer clarity in the current discourse on vaccines in the wake of COVID‐19. Moreover, this study further contributes to current discourse on the COVID‐19 pandemic (Callaghan et al., 2021; Durizzo, Asiedu, Van der Merwe, Van Niekerk, & Günther, 2021; Dhanani & Franz, 2021; Park & Chung, 2021; Latkin, Dayton, Yi, Konstantopoulos, & Boodram, 2021; Sheng et al., 2021) by integrating COVID‐19 insights to advance a novel business and public policy research agenda with the aim of meeting some of the new global challenges. In addition, although past studies on the COVID‐19 pandemic have illuminated understanding of governments' responses (e.g., Mintrom & O'Connor, 2020), there is a paucity of research on “vaccine nationalism” and its implication for international business research. This study examines this issue and outlines avenues for future research.

In the remainder of this article, a brief overview of the COVID‐19 pandemic and vaccine distribution strategies is presented. This is followed by the development of a conceptual framework and then an outlining of a new agenda for future research is presented.

## 2 COVID‐19 AND VACCINE DISTRIBUTION STRATEGIES: AN OVERVIEW AND CONCEPTUALIZATION

In the past few years of COVID‐19 pandemic, healthcare systems in many developed and emerging nations have been teetering toward collapse and unable to handle the growing hospitalizations (Drexler, 2021). The financial and economic shock has cascaded into many firms fighting for survival (Amankwah‐Amoah et al., 2020a). Indeed, countries' financial resources have been severely depleted as a result of having to divert funding to combatting the disease and helping small businesses stay buoyant (Bartik, Bertrand, Cullen, Glaeser, Luca, & Stanton, 2020).

Following the dislocation of voluminous small businesses across the globe, governments have increasingly been called upon to shore up such firms to be able to transition to the future. For instance, in the US, the government partly achieved this objective via the Coronavirus Aid, Relief, and Economic Security Act (CARES Act) (Bartik et al., 2020). Although some resource‐constrained nations have better weathered the coronavirus pandemic such as Bhutan and Vietnam (Drexler, 2021), developed nations continue to struggle to maintain effective handling of the crisis.

### 2.1 Businesses and COVID‐19 vaccine development

Owing to the native effects of lockdown measures, border closures, and cessation of businesses, governments and donor/philanthropic organizations such as the Gates Foundation sought to boost investment in developing vaccines to help find solutions and support national economies to recover in a speedy manner (Hooker & Palumbo, 2020). Indeed, governments around the globe have provided over £6bn and £1bn from donor/philanthropic organizations toward vaccine development (Hooker & Palumbo, 2020). Accompanying the pandemic has been a massive investment by pharmaceutical companies into vaccine development activities. For instance, the US firm Johnson & Johnson worked in collaboration with the UK's AstraZeneca/University of Oxford‐based biotech company to develop one of the vaccines (Hooker & Palumbo, 2020). In a similar vein, Moderna, a biotechnology firm, has also worked with not‐for‐profit organizations in bringing its vaccine to market. At the core, the pharmaceutical industry has responded to the crisis by prioritizing research and development activities related to COVID‐19 and new technology adoption to underpin drug development (Association of the British Pharmaceutical Industry, 2021). It is worth noting that some of the pharmaceutical companies have also charged different amounts in different countries, which might benefit developing nations (Hooker & Palumbo, 2020). Despite substantial resource investment, the pharmaceutical company Merck shelved its two COVID‐19 vaccine candidates—known as V590 and V591—due to unfavorable results from clinical trials (Chappell, 2021b).

Pharmaceutical companies have also mobilized a range of new technologies to disseminate not only information but also to minimize face‐to‐face interaction to curtail contagion mechanisms for the virus. This also means the increasing use of virtual teams as a means of managing their activities. Indeed, modern ICT offers ample opportunities for R&D activities, at any global location of the firms, to forge international partnerships and disseminate knowledge (Castellano, Chandavimol, Khelladi, & Orhan, 2021). Virtual research and development (R&D) teams help to maintain a virtual presence to foster an effective functioning of the team (Castellano et al., 2021). A major challenge stemming from COVID‐19 is the obligations imposed on businesses regarding health and safety (Manuel & Herron, 2020). Indeed, numerous organizations have not only instituted new cleaning regimes but also offer personal protective equipment (PPE) at work, encompassing items, such as gloves, eye protection, face shields, clothing, and safety wear.

Although facilitating knowledge diffusion can be challenging in virtual teams due to potential misinterpretation of signals, the cost of transmitting information and communication has shrunk (see Klitmøller & Lauring, 2013). One possible explanation is that technologies, such as video conferencing, Zoom and Teams offer opportunities to minimize face‐to‐face interactions. By avoiding face‐to‐face meetings and requiring limited office space to work and organize firms' activities, organizations are able to conserve financial resources. During the COVID‐19 pandemic, environmental sustainability issues and practices have also been elevated to the forefront of businesses' agenda (Amankwah‐Amoah, 2020a, 2020b), focusing on the reduction in greenhouse gas emissions, air pollution, carbon emission footprints, and energy consumption (Chowdhury, Paul, Kaisar, & Moktadir, 2021).

## 3 GLOBAL VACCINE DISTRIBUTION STRATEGIES

To deduce the patterns related to global COVID‐19 vaccine distribution strategies, the study relied mainly on secondary/archival sources. To mobilize information for the conceptualizations, this article utilized insights from COVID‐19‐related press releases and reports from international organizations data such as the European Commission, OECD, US Food and Drug Administration and World Health Organization. In addition, industry/business periodicals (e.g., The Economist and Airline Business) alongside news reports (e.g., BBC, CNN and The New York Times Company) were also consulted.

**FIGURE 1:**
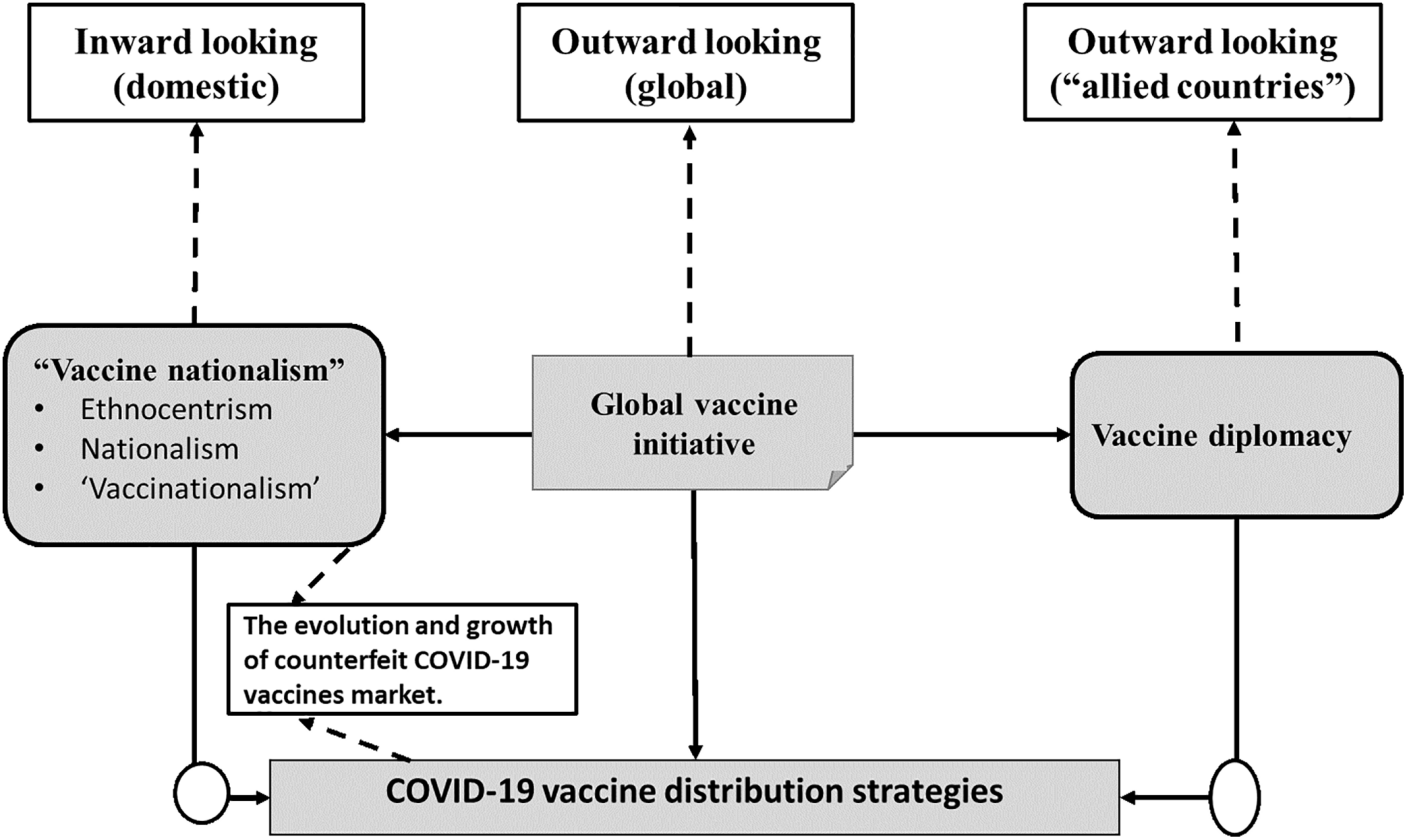
Global COVID‐19 vaccine distribution strategies.

Globally, governments including US, UK, and China have played a vital role in providing financial resources to pharmaceutical companies not only for COVID‐19 vaccine development but also its distribution. For instance, in the US, the government has used the Defense Production Act, a wartime power to obtain equipment and accelerate vaccine manufacturing (Lupkin, 2021a). By invoking the Defense Production Act 2021, the US government was able to leverage its powers and resources to get pharmaceutical giant Merck to help manufacture Johnson & Johnson's COVID‐19 vaccine (Lupkin, 2021b). This was a major step given that the two companies are rivals and historically opted to compete rather than collaborate. Although the development of COVID‐19 vaccines in record time is regarded as one of the greatest achievements of scientists in modern times (Ghebreyesus, 2021), the distribution of vaccines has been marked by divergent approaches. The research note (RN) conceptualizes the global vaccine distribution strategies into broadly three perspectives (i.e., “vaccine nationalism,” vaccine diplomacy, and global vaccine initiative), as demonstrated in Figure 1.

Table 1 summarizes some of the various COVID‐19 vaccines in use. This table also demonstrates that governments, including the United States, Britain, and members of the EU have played an active role in vaccine development in terms of investment in research and development‐related activities.

**TABLE 1:** Summary of some COVID‐19 vaccines authorized for use

Vaccine, developers/ sponsors/pharmaceutical companies	Country of origin	Role of government and other stakeholders	Brief description
BBIBP‐CorV Sinopharm	China	Support to the pharmaceutical firm.	Effective and offers protection against COVID‐19.
Sputnik V COVID‐19 vaccine	Russia	Supported by governments in terms of financial resources.	Effective and offers protection against COVID‐19.
Pfizer‐BioNTech COVID‐19 vaccine	United States and Germany	Supported by governments in terms of financial resources.	Effective and offers protection against COVID‐19.
Moderna COVID‐19 vaccine	United States	Supported by governments in terms of financial resources.	Effective and offers protection against COVID‐19.
Oxford university –AstraZeneca COVID‐19 vaccine	United Kingdom	Government investment in the research and development‐related activities.	Effective in combating COVID‐19.
Janssen Ad26.COV2.S vaccine, Johnson & Johnson	Netherlands and United States	Supported by governments.	The ability for it to be stored in a refrigerator rather than a freezer partly makes it a cost‐effective alternative to the Pfizer and Moderna vaccines.

*Note*: *Data sources*: synthesized by the authors from multiple sources including: World Health Organization, 2022; US Food and Drug Administration, 2021; Jennings, 2021; GAVI, 2021; Chappell, 2021a, 2021b.

### 3.1 Vaccine nationalism

The first of the global vaccine distribution strategies is “vaccine nationalism.” According to Evenett et al. (2021, p. 1), “vaccine nationalism can take the form of overt export bans or limits—that aim at increasing domestic availability of vaccines at the expense of foreign supply—or they can take less transparent but often equally effective forms.” This is rooted in weak or lack of cooperation and collaboration between nations to facilitate timely and equal access to vaccines. A defining characteristic of this perspective is its inward/domestic focus on vaccine distribution permanently or temporarily and also in terms of producing nations creating trade barriers or even delays in shipments.

As previously noted, the global economy is set to lose around $9.2 trillion if “vaccine nationalism” persists, thereby leaving the developing world behind in inoculating their citizens (Busari & Cheung, 2021). Even if all citizens of developed economies are vaccinated, the potential for a new version of the virus to re‐merge could linger. Although around 85% of developing nations are yet to have effective vaccination programs and “there is a growing realization that the virus is likely to find a permanent home in humans,” developed nations have accelerated their programs (The Economist, 2021a, p. 9–10). Indeed, prior to an effective vaccine emerging, the World Health Organization Director‐General Tedros Adhanom Ghebreyesus urged nations to focus on global access to mitigate “hoarding” by some leading nations' “vaccine nationalism” (Neuman, 2020a; 2020b).

Another feature of vaccine nationalism is a tendency among governments to seek to secure vaccine doses to the detriment of others (Evenett, Hoekman, Rocha, & Ruta, 2021). For instance, in March 2021, the European Union and Italy withheld a shipment of 250,000 AstraZeneca vaccine doses which were bound for Australia with the aim of ensuring that their domestic needs were catered for first (The Economist, 2021b). This partly stems from the new European Union requirements (i.e., *European Commission Implementing Regulation* 2021/111), which imposed on companies the requirement to seek permission for export of COVID‐19 vaccine or vaccine components from both the individual nation where manufacturing is undertaken as well as the European Commission (European Union, 2021; The Economist, 2021b; Evenett, 2021). This was designed with the aim of addressing a perceived lack of transparency of COVID‐19 vaccine exports outside the EU while concurrently ensuring timely access to vaccines among all EU members (European Union, 2021). In a similar vein, the US and British governments have also reached agreement with manufacturers that “locked up domestic output for their citizens” culminating in “stockpiles” (The Economist, 2021b, p. nd). These new waves of trade restrictions have potential to curtail the flow of goods in a post‐pandemic environment. Another way these kinds of trade restrictions have manifested is in the export curbs on personal protective equipment (PPE) during the early phase of the pandemic. Indeed, according to Global Trade Alert, there are up to 137 export curbs on PPE and other medical products applied in more than 70 jurisdictions (The Economist, 2021b).

However, the notion of “vaccine nationalism” is a recipe for greater ethnocentrism on the global stage where more and more consumers/citizens develop a hostile attitude toward foreign‐made products. The term ethnocentrism is used in a sense that it can act as a “self‐defense reflex of local economies, governments, organizations, and individuals against the threat of imports and foreign competition” (Siamagka & Balabanis, 2015, p. 66). Besides just government encouragement to buy homemade products, the elevation of domestic priorities over all concerns is worrying. The forces of globalization face a headwind due to some countries becoming more inward looking and nationalistic. The “vaccine nationalism” approach was adopted by many western nations including the US and European nations. The necessity of vaccines in re‐starting national economies after lockdowns around the globe appears to have created an incentive for some governments to initiate and implement some forms of protectionist measures.

### 3.2 Vaccine diplomacy

In contrast to the above approach, vaccine diplomacy refers to a “branch of global health diplomacy that relies on the use or delivery of vaccines” (Hotez, 2014, p. 1). A distinctive characteristic of this perspective is its focus on humanitarian intervention as a means of enhancing a country's standing. Under the umbrella of vaccine diplomacy, is vaccine science diplomacy, which refers “to the joint development of life‐saving vaccines and related technologies, with the major actors typically scientists” (Hotez, 2014, p. 2). For instance, China, India, and Russia have championed the so‐called “vaccine diplomacy” as means of amassing goodwill in the developing world and developing “soft power” (Myre, 2020). To further elucidate, Russia has made its Sputnik V COVID‐19 vaccine, which offered a robust protective effect, widely available to developing nations and this has helped in this direction (Jones & Roy, 2021). In the meantime, China has also spent around $2 billion to help developing nations as well as making its vaccine widely available (Myre, 2020). By February 2021, around a million doses of China's Sinopharm COVID‐19 vaccine had been exported to countries, such as Nepal, Pakistan, Cambodia, Sierra Leone, and Zimbabwe as a key pillar of its “vaccine diplomacy” strategy (Jennings, 2021).

At another level, India has donated some supplies of the AstraZeneca/Oxford vaccine, which is made in the country, to neighboring countries such as Bangladesh, Myanmar, and Nepal (Jennings, 2021). “Vaccine diplomacy” may also reverse or halt the global economic integration and interdependence of nations to facilitate cross‐border flow of capital, goods, and services. The uneven distribution of COVID‐19 vaccines can manifest as “advanced economies are tightly connected to unvaccinated trading partners which consist of a large number of emerging markets and developing economies” (Blanchfield, 2021, p. nd). In over three decades, scholars have sought to offer a fresh perspective on the “new” Africa–China relationship and China's noninterventionist approach. Vaccine nationalism can be seen as part and parcel of this approach. Accompanying this perspective on vaccine nationalism, China in particular, places the emphasis on different global engagement to foster collaboration influence allies in Africa. On other hand, the new relationship rooted in “vaccine nationalism” can be viewed as a new form of colonialism with potential to have adverse consequences for African countries by creating new sources of dependences on China.

### 3.3 Global vaccine initiative

The final perspective is the global initiative with multilateral collaboration to effectively combat the global pandemic. COVAX is spearheaded by multiple parties including the Coalition for Epidemic Preparedness Innovations, GAVI, UNICEF, and the World Health Organization with global risk‐sharing focus (GAVI, 2021; Neuman, 2020a; 2020b) and seeks to deliver 2 billion vaccine doses globally by the end of 2021 (BBC, 2021b). The emphasis on greater and equitable access to COVID‐19 vaccines is championed by the organization, and also assigns credit to international organizations such as the World Health Organization and UNICEF, and away from individual countries. By focusing coalition building, the approach has the potential to create a more harmonious global environment. Thus, far the COVAX program has accumulated $6bn funds with support from countries, such as the US, UK, and France, and seeks to deliver over 2 billion doses to people in 190 nations within a year (BBC, 2021).

In late February 2021, Ghana, a West African country with a population of more than 30 million, became the first country in the world to receive coronavirus vaccines under the COVAX vaccine‐sharing initiative (BBC, 2021b; Busari & Cheung, 2021). The batch delivered included 600,000 doses of the AstraZeneca vaccine, thereby taking a giant step in meeting COVAX's objective of helping to bridge the gap between advanced countries and developing nations in accessing the vaccines (BBC, 2021b). This was to be followed by delivering to other countries in the region and beyond. By focusing on international effort geared toward helping low‐ and middle‐income countries vaccinate their citizens and cope with the pandemic (Chappell, 2021a), the COVAX global program represents some kind of mid‐point between the “vaccine nationalism” and vaccine diplomacy approaches adopted on the global stage. Rooted in the above analysis, Table 2 summarizes the merits and demerits of the three competing perspectives.

**TABLE 2:** COVID‐19 pandemic and global vaccine distribution strategies

Competing perspectives	Merits	Demerits
“Vaccine nationalism”	Inward attitude to vaccine distribution. Can foment intense quest for resources between nations. Largely designed to focus on vaccination requirements in the “motherland” for a period. Ignites the spirit of “patriotism” in either vaccine development or distribution, or both. Seen to counter the growing tide of globalization around the globe.	Nationalism can lead to ethnocentrism, which can manifest in citizens and consumers' behavior. Hoarding by some countries can undermine harmony and trust between nations. Potential to undercut international collaboration between countries. Forces weaker and resource‐poor nations to become wearier of rich and developed nations. Potential to exacerbate nationalism tendencies.
Vaccine diplomacy	Leveraging vaccine distribution to gain goodwill and improve a country's standing around the globe. Home‐ and allied‐countries' first approach.	It has potential to elevate less democratic nations, such as Russia and China on the global stage. Vaccine diplomacy could encourage developing nations to reduce their dependence on developed nations for vital suppliers beyond vaccines.
Global vaccine initiative	Recognized the globalization realities and benefits of global access to vaccines. A vital ingredient for effective multilateral cooperation and all‐countries first approaches.	Domestic citizens may resent the notion of elevating other countries' citizens' concerns and access to vaccines to the top of global priority.

## 4 COVID‐19 AND COUNTERFEIT VACCINES

Broadly speaking, around the globe, over 2 billion people do not have “access to necessary medicines, vaccines, medical devices, including in vitro diagnostics, and other health products” (World Health Organization, 2021b, p. nd). This issue is particularly important given that prior to COVID‐19, the World Health Organization observed that 1 in 10 medical products in low‐ and middle‐income nations were substandard/falsified (WHO, 2018). According to Research and Markets' report (2018), counterfeiting products are valued around 1.2 trillion USD in 2017. In Africa, for instance, around 18.7% medicines are either substandard or falsified (Ozawa et al., 2018). This preexisting trend has been further amplified by the global unequal access to vaccines.

Following the preceding analysis, one of the outcomes of the unequal distribution of vaccines has been the spread of counterfeits COVID vaccines (see Amankwah‐Amoah, 2022). Some contend that vaccine “hoarding” by advanced nations has not only created conditions for “vaccine nationalism” (Neuman, 2020a; 2020b), but also created the conditions forcing many developing nations and citizens to explore other means of accessing the vaccines. According to World Health Organization (2022), equitable access to safe and effective vaccines is sine qua non in countries' ability to combat and return the world to greater degree of normalcy. Despite this important recognition, “vaccine nationalism” remains a major issue.

In the vacuum of lack of access to vaccines and prevalence of fake information, many questionable products have also emerged in the “marketplace” in both developed and developing nations purporting to help diagnose, remedy, treat, and even prevent COVID‐19 disease (US Food and Drug Administration, 2022; INTERPOL, 2020). In the midst of that desperation, counterfeiters and illegitimate business have flourished by offering unproven products to citizens claiming to cure the coronavirus. One of the downsides of the spread of such products is that its disincentives drug manufacturers from investing in new products. Another important side effect is That such substandard and falsified COVID medicines can lead to serious and life‐threatening harm (US Food and Drug Administration, 2022).

### 4.1 Vaccine passport

There is anecdotal evidence suggesting that vaccine passports offer an effective pathway to return industries and the world back to normalcy (see Boon, 2021; European Commission, 2021; IATA, 2022). Broadly speaking, the vaccine passports is some kind of “immunization certificates” encompassing not vaccination status of the holder, but also results from infection tests, proof that the individual possess a “completed a period of quarantine, or exemptions from vaccination for health reasons” (The Economist, 2021c, p. 75–77). For instance, the European Union, the European Commission' plan has been put in motion for the so‐called “Digital Green Certificate” and thereby offering verifiable vaccination status of individuals (European Commission, 2021; The Economist, 2021c). The certificate seeks to create conditions that help to allow free intra‐EU movement during the pandemic (European Commission, 2021). Nevertheless, distinguishing and offering different treatments and opportunities for travel and engagement in economic activities for vaccinated and unvaccinated individuals in a world with unequal access to vaccines is a recipe for disaster.

Around the globe, many organizations also require workers to demonstrate proof of proof of vaccinations and thereby leading to the growth of fake vaccination cards/ID. Although vaccine passport is not new as travelers have historically been advised to pre‐vaccinate prior to traveling to certain regions, a vita question that remains underexplored is how the dynamics of vaccine passport on how businesses formulate their strategies and organize themselves.

## 5 GENERAL DISCUSSION AND IMPLICATIONS

Drawing on insights on the COVID‐19 pandemic, this research note examined the global vaccine distribution strategies. The article conceptualized the global vaccine distribution strategies into three competing perspectives (i.e., “vaccine nationalism,” vaccine diplomacy, and global initiative) that capture the global dynamics related to vaccine rollout. By critically examining the COVID‐19 pandemic, the study shed light on potential implications for businesses and advanced a fresh research agenda which reflected the modern realities of vaccine distribution. Although there have been notable studies of vaccine distribution related to COVID‐19 (Rastegar, Tavana, Meraj & Mina, 2021), lacking in the current discourse is how “vaccine nationalism” and vaccine diplomacy could impact on governments and businesses originating from countries with the divergent approaches. In light of growing scholarly interest in the COVID‐19 pandemic, the study further contributes to the literature by fostering a deeper understanding and implications of “vaccine nationalism” and vaccine diplomacy. Guided by the emerging research themes stemming from the COVID‐19 pandemic and vaccine rollout, this article outlines a number of research challenges and opportunities for advancing contemporary research.

### 5.1 Theoretical and practical contributions

The analysis yielded several important contributions to theory and practice. Theoretically, prior scholarly efforts neither theoretically nor empirically have sought to elucidate and differentiate the divergent global vaccine distribution strategies during the pandemic (e.g., Fernandes et al., 2022; Santos et al., 2021). In addressing this lacuna in our understanding, the study developed a conceptual model of the approaches and provide deep insights into this issue. Given the increasing importance of vaccine distribution as a key pillar in global economic recovery from the pandemic along with the growing necessity to protect public health, our investigation builds on the body of research and public discourse on the subject by focusing on the wider implications. The analysis contributes to the current literature by speaking to the ongoing discourse surrounding the COVID‐19 pandemic and business, where China has pursued vaccine diplomacy with the aim of aiding developing nations.

Viewing quality and attentive leadership as essential ingredients in not only identifying and responding to the crisis (Drexler, 2021), it is contended that global leadership would be crucial in the post‐pandemic phase. The analysis also suggests a need for global organizations, such as WHO and UN to move toward de‐escalating the forces driving “vaccine nationalism” and move toward a more global solidarity approach that fosters harmony. This would go a long way in ushering in a post‐pandemic global economic recovery rather than regional recovery. The potential for heightening nationalism and ethnocentrism is real with regard to the “vaccine nationalism” approach which must be borne in mind. Thus, more resources need to be geared toward COVAX to quell this. Given that vaccine distribution is seen as the best chance of ushering in global economic recovery, it is incumbent on governments and policymakers to ensure that they do not succumb to nationalism and vaccine nationalism tendencies. By encouraging nations to join forces in scaling‐up of COVID‐19 vaccine production and distribution has the potential of reducing tension and potential global conflicts between countries.

### 5.2 Limitations and future research directions

There are several limitations which culminate in the direction for future research. First of all, a limitation is that the analysis provides a snapshot on an unfolding event with consequences for years to come. Thus, future study could seek to extend our insights into the effects of COVID‐19 in the post‐pandemic business landscape. Future research could build on prior efforts by investigating how global variation in access to COVID‐19 vaccine can be traced to the quality of a country's informal and formal institutions, such as legal system, culture, and beliefs. Pertaining to this, the specific questions for future research are illustrated in Table 3. The empirical and practitioner literature has been contradictory with regard to long‐term effects of vaccine diplomacy. Thus, a more robust analysis of the long‐term implications for governments and how businesses formulate their strategies in response to vaccine nationalization and diplomacy is needed.

**TABLE 3:** COVID‐19 pandemic and emerging research themes

Key themes	Characterization	Emerging research opportunities and vital future questions
“Vaccine nationalism”	Home‐country first approach.	What firms organize their activities in the wake of COVID‐19‐induced disruptions to reduce reliance on some countries? How do geographically dispersed co‐workers or teams benefit from “vaccine nationalism”? To what extent does the “home‐country first approach” lead to limited international engagement by businesses?
Vaccine diplomacy	Leveraging vaccine distribution to win. Home‐ and allied‐countries first approach.	To what extent can “vaccine diplomacy” undermine the “rules of the game” governing developed and developing nations' firms on the global stage? Can “vaccine diplomacy” be turned into a source of market competitiveness for emerging market firms from countries such as China and Russia? Can “vaccine diplomacy” have a knock‐on effect in fostering greater technology transfer to developing economies?
Global vaccine initiative	Multilateral cooperation and all‐countries first approaches. Global access to vaccines.	How do businesses organize their global activities to provide pandemic security? Would the pandemic make firms and governments more environmentally aware of future challenges? To what extent can the variations in access to COVID‐19 vaccine be traced to the quality of a country's informal and formal institutions such as legal system, norm, culture, and beliefs? What would be the effects of COVID‐19 in nurturing and creating cross‐border and supply‐chain partnerships? To what extent do governments and other stakeholders capitalize on the COVID‐19 pandemic to champion stringent sustainable practices for MNEs and SMEs? What are the opportunities and potent incentive measures to usher in a greater green revolution in this new era?

*Note*: *Data sources*: synthesized by the authors from multiple sources including Callaghan et al., 2021; Amankwah‐Amoah et al., 2022; Dhanani & Franz, 2021; Amankwah‐Amoah, 2021; Amankwah‐Amoah et al., 2021; Latkin et al., 2021.

In this study, we conceptualized three perspectives around the globe (i.e., “vaccine nationalism,” vaccine diplomacy, and global initiative) and their rationale. Future research could explore which of these approaches offer a more conducive environment for start‐up domestic business' development. One possible line of thinking is that “vaccine nationalism” might be effective for the development and scaling‐up of new domestic businesses, given the “protection” offered to such organizations. Unlike firms forged under vaccine diplomacy, and global initiative it has the potential of creating conditions for development of businesses that are unlikely to be competitive in a global environment. Although the global supply of COVID‐19 vaccine doses is surging, the initial roots of nationalism and diplomacy have been planted, which can have effects on nations beyond the pandemic.
